# Gold Nanoparticles Enhance Radiosensitivity in Glioblastoma Cells

**DOI:** 10.1021/acsomega.6c00540

**Published:** 2026-03-17

**Authors:** Laura Coppola, Giovanna Navarra, Giorgio Avilia, Carlos Cuestas-Ayllón, María Moros, Jesús Martínez de la Fuente, Pasqualino De Antonellis, Maria Teresa Gentile, Laura Mosca, Caterina Oliviero, Roberto Pacelli, Chiara Laezza, Maurizio Bifulco, Cristina Pagano

**Affiliations:** † Department of Molecular Medicine and Medical Biotechnology, 9307 University of Naples “Federico II”, Naples 80131, Italy; ‡ Instituto de Nanociencia y Materiales de Aragón (INMA), 82976 CSIC-Universidad de Zaragoza, 50009, Zaragoza, Spain; § Centro de Investigación Biomédica en Red de Bioingeniería, Biomateriales y Nanomedicina (CIBER-BBN), 28029, Madrid, Spain; ∥ Department of Environmental, Biological and Pharmaceutical Sciences and Technologies (DiSTABiF), 18994 University of Campania “Luigi Vanvitelli”, Caserta 81100, Italy; ⊥ Department of Human Sciences and Promotion of the Quality of Life, San Raffaele University, Rome 00166, Italy; # Unit of Medical Physics and Radioprotection, University Hospital Federico II, Naples 80131, Italy; ¶ Department of Advanced Biomedical Sciences, University Federico II, Naples 80131, Italy; ∇ Institute of Endocrinology and Experimental Oncology (IEOS), National Research Council (CNR), Naples 80131, Italy

## Abstract

Background: Glioblastoma multiforme (GBM) is a highly aggressive primary brain tumor with a poor patient prognosis. Standard of care treatment includes maximal surgical resection, followed by radiotherapy and temozolomide administration. Despite this multimodal strategy, patients fail to achieve an effective response due to resistance to radio-treatment, allowing the tumor to recur. Therefore, there is a compelling need to discover new radiosensitizer approaches to overcome radioresistance and improve the response and survival of patients affected by glioblastoma. A novel approach is represented by nanoradiosensitizers, such as gold nanoparticles (AuNPs), able to enhance the effectiveness of radiotherapy. Methods: In this study, we assessed the biological interaction of AuNPs with ionizing radiation (IR) in both immortalized and primary patient-derived glioblastoma cell lines. Results: we observed that the combinatory effect of AuNPs with IR decreased cell viability and increased necrosis and apoptosis compared to cells treated only with IR or untreated cells. Additionally, our results showed an increase in the sensitization enhancement ratio (SER) of cells treated with AuNPs and IR. Furthermore, AuNPs showed a tumor-specific effect, since it did not seem to support the effects of radiation on the normal human astrocyte (NHA) cell line. Conclusions: Our results suggest that the use of AuNPrs can improve radiotherapy efficacy by increasing the radiosensitivity of the targeted cells.

## Graphical abstract

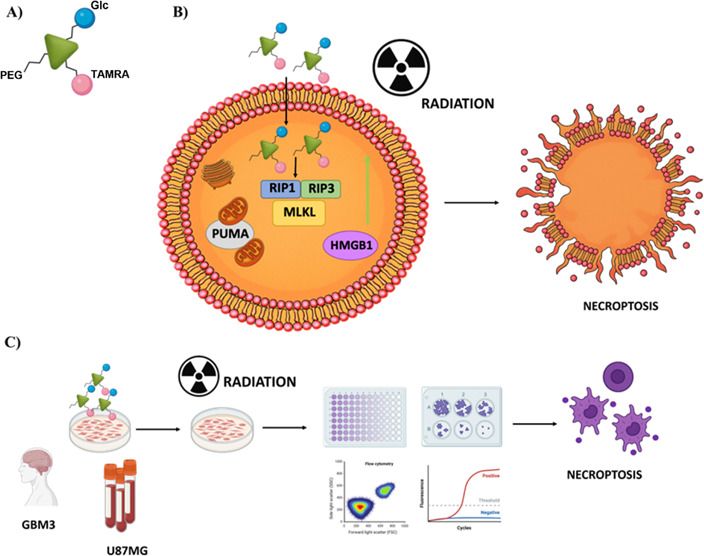

## Introduction

Glioblastoma multiforme (GBM), a grade IV astrocytoma, is the most common and aggressive primary brain tumor found in adults. GBM is characterized by short survival times (<15 months) and unfavorable prognosis due to limited response to available therapies such as radiotherapy (RT) and chemotherapy.^1^

RT treatment occurs at very high doses, up to 56–60 Gy, with standard fractionation (2 Gy/day).^2,3^ This treatment is based on the DNA damage caused by the exposure of biological tissues to ionizing radiation (IR) and has shown several limitations due to toxic side effects, including heterogeneity in dose distribution and prolonged exposure of healthy tissues. There is strong evidence suggesting that GBM develops radioresistance (RR), demanding higher radiation doses for treatment, which is the main reason for the high mortality rate of affected patients.^4^ Resistance to RT is due to tumor heterogeneity and various biological mechanisms such as altered cell cycle, DNA damage, inflammation, and the presence of glioblastoma stem cells (GSCs) that promote tumor progression.^5^ These cells can survive exogenous DNA damage, due to robust DNA repair, and repopulate the tumor following treatment, thereby contributing to RR and tumor recurrence.^5^ Different approaches have been proposed to overcome RR of GBM, divided into two main categories: implementation of advanced RT techniques and development of a new generation of agents that sensitize cells to IR. Since GBM is known to be resistant to radiation and often recurs in the area that was treated with RT, tumor radiosensitization represents a key strategy to improve the clinical outcome in GBM patients. Therefore, compounds that improve the effective dose of RT in tumor cells have gained significant interest. Specifically, high-atomic-number (*Z*) nanomaterials, are commonly used as dose enhancers for RT, due to their strong photon attenuation and capacity to enhance radiation dose deposition.^6^ Numerous experimental data have confirmed the radiosensitization potential of gold (*Z* = 79) nanoparticles (AuNPs). They are promising radiosensitizers due to their size, high stability, biocompatibility, and the ability to be functionalized with different biomolecules that can increase their targeting ability toward tumoral cells for instance. These characteristics can influence their biodistribution, enhancing their accumulation in tumor tissue, which could in turn generate high local ionization, decreasing both treatment duration and radiation doses. Thus, healthy tissues absorb a reduced dose of radiation, leading to fewer side effects caused by RT.^7^ In this study, we used a type of anisotropic gold nanoparticles, nanoprism (AuNPrs), functionalized with polyethylene glycol (PEG). PEG is a biopolymer that prevents immune recognition, stabilizes the nanoparticles, and reduces their toxicity. Moreover, AuNPrs are conjugated with molecules of glucose, chosen to specifically target cancer cells that overexpress glucose receptors,^8^ and with a fluorophore, to verify the localization of the nanoparticles in the cells.^9,10^ AuNPrs offer several advantages over other types of gold nanoparticles. They can be synthesized through a simple, solvent-free method and are excitable at wavelengths ranging from 700 to 1200 nm. Upon excitation, they convert the absorbed energy into heat, destroying cancer cells or disaggregating protein aggregates, a phenomenon known as the photothermal effect. In this study, we employed AuNPr as a radiosensitizer for the first time. We analyzed their effect on the viability and radiosensitivity of the U87MG immortalized cell line and of a GBM primary cell line, here named GBM3, directly isolated from an affected patient.

## Materials and Methods

### Cell Cultures

Normal Human Astrocytes (NHA) were cultured in astrocyte basal medium (ABM) supplemented with the astrocyte growth medium AGM SingleQuots Kit (Lonza). The U87MG cell line was purchased from CLS Cell Lines Service GmbH (Eppelheim, Germany) and maintained in Dulbecco’s modified Eagle’s medium (DMEM) supplemented with 10% heat-inactivated fetal bovine serum (FBS), 1% sodium pyruvate, 1% l-glutamine, 1% non-essential amino acids (Lonza, Italy), 1% penicillin–streptomycin solution, and 0.1% Plasmocin prophylactic (InvivoGen). All cell cultures were maintained at 37 °C in a humidified 5% CO_2_ atmosphere.

### Preparation of the Glioblastoma Primary Cell Line

Tumor tissue was collected during surgery at the Neurosurgery Service of “Antonio Cardarelli” Medical Hospital (Naples, Italy) following the Institutional Committee Ethical Standards (DEL. N°897, August 13, 2020). Primary tumor cell lines were established from tumor biopsies; a second specimen from each biopsy was reserved for clinical evaluation according to the WHO guidelines. All participants provided written informed consent. Briefly, the adherent primary culture here designated as GBM3 was prepared starting from the mincing and dissociation of tumor tissue using the gentleMACS Dissociator and Tumor Dissociation Kit (Miltenyi Biotec, code # 130-095-929). The cell suspension was filtered, washed with RPMI-1640 (Microtech), and centrifuged before being cultured in DMEM/Ham’s F-12 (Gibco) medium supplemented with 15% heat-inactivated fetal bovine serum (FBS), 1% sodium pyruvate, 2% l-glutamine, 1% non-essential amino acids (Lonza), 1.5% d-glucose, and 0.1% Plasmocin prophylactic (InvivoGen). The cells were cultured at 37 °C in a humidified 5% CO_2_ atmosphere.

### Primary Glioblastoma Characterization

DNA methylation across 850,000 CpG sites per tumor sample was assessed using the Illumina EPIC ARRAY 850 K beads-chip performed following the manufacturer’s instructions. A specialized algorithm and a bioinformatics package were used to compare the epigenomic profile to a reference cohort from the German Cancer Research Center. Array data were also analyzed to generate copy number variation profiles, which were used to assess the presence/absence of the EGFR gene.^11^

### Synthesis and Characterization of AuNPrs

The synthesis of gold nanoprisms (AuNPrs) was optimized based on a previously reported protocol. Briefly, 120 mL of 0.5 mM Na_2_S_2_O_3_ (containing 0.1 M KI) was slowly added to 100 mL of 2 mM aqueous HAuCl_4_. After 4 min without stirring, a second identical addition was made. Finally, an additional 100 mL of 0.5 mM Na_2_S_2_O_3_ was added. The reaction was then allowed to continue for 1 h at room temperature without agitation. The resulting AuNPrs were subsequently functionalized with HS-PEG-COOH (5000 g/mol) at mass ratios of NPrs:PEG of either 1:2. NaBH_4_ was added in a 1:1 molar ratio relative to PEG, and the pH of the solution was adjusted to 12 using 2 M NaOH. The mixture was then subjected to ultrasonication at 60 °C for 1 h. Unreacted reagents were removed by centrifugation at 5000*g* for 15 min (three cycles), and the resulting pellets were resuspended in Milli-Q water. Although the synthesis aimed to produce AuNPrs, spherical gold nanoparticles were also generated as byproducts, exhibiting localized surface plasmon resonance (LSPR) peaks at 540 nm. To purify the AuNPrs, agarose gel electrophoresis was performed by using a customized system with 12 enlarged sample wells, each loaded with 2000 μL of the reaction mixture. The agarose gel (2.5% w/v) was prepared in 0.5× TBE buffer (Merck Millipore, MA, USA) using a microwave. Prior to loading, a 25% (v/v) glycerol solution in 0.5× TBE was added to the reaction mixture to increase density. Specifically, 10 mL of the reaction mixture was combined with 1.8 mL of glycerol solution and loaded into the wells. Electrophoresis was carried out at 140 V for 30 min, enabling spherical AuNPs and smaller AuNPrs to migrate into the gel matrix, while the purified AuNPrs remained at the loading zone and were subsequently collected. The purified AuNPrs were washed via centrifugation with Milli-Q water, and their final concentration was determined by UV–vis spectroscopy, using an experimental absorbance coefficient (ε) of 35.86 mL mg^–1^ cm^–1^. The average edge length of the AuNPrs was 277 ± 36 nm, as determined by scanning electron microscopy (SEM, Inspect F50 equipped with a system EDX INCA PentaFET x 3, FEI company, Hillsboro, Oregon, USA). Their LSPR spectrum, measured by a UV–vis spectrophotometer (Jasco V-670, Jasco Corporation, Tokyo, Japan), showed a near-infrared (NIR) peak centered at 1100 nm.

### Biofunctionalization with TAMRA and Glucose

Afterward, PEG-derivatized AuNPrs were functionalized using amine-modified glucose. Briefly, 0.25 mg of PEG-derivatized AuNPrs were incubated with 3 mM EDC and 7 mM Sulfo-NHS in 0.5 mL of filtered 10 mM MES buffer pH 6 for 30 min at 37 °C. After that the activated AuNPrs were centrifuged 9 min at 6500*g*. After removal of the supernatant, the AuNPrs were dispersed with 24 nmol of 5(6)-TAMRA cadaverine diluted in 0.5 mL of filtered 50 mM MES buffer pH 6 and incubated for 1.5 h at 37 °C. Then, 0.5 mL of 4-aminophenyl-ß-d-glucopyranoside (7400 nmol) in filtered 50 mM MES buffer (pH 6.0) was added to the AuNPrs solution, incubated for 2 h at 37 °C, and added to derivatize the remaining activated carboxylic groups. Finally, biofunctionalized AuNPrs were washed out of excess ligand by centrifugation three times for 9 min at 6500*g*, and then, pellets were resuspended in filtered 10 mM HEPES buffer pH 7.2 containing 0.1% Tween 20 and 0.1% BSA.

### MTT Assay

GBM cells were seeded at a density of 1 × 10^3^ cells/well in 96-well plates. AuNPrs were administered in increasing concentrations (0.1–0.8 mg/mL). After 24 h of treatment, the cells were incubated for 4 h with 0.5 mg/mL MTT (3-[4,5-dimethylthiazol-2-yl]-2,5 diphenyl tetrazolium bromide) (Sigma-Aldrich) to allow the formation of purple formazan crystals. The crystals were solubilized using acid-isopropyl alcohol (10% HCl 1N in isopropanol). Absorbance was then measured at 570 nm using a Synergy HT Microplate Reader (BioTek Instruments, Inc.) to assess cell viability. The same MTT protocol was used to evaluate the effects of combined AuNPrs treatment and radiation exposure (2 Gy, 4 Gy, 8 Gy) over 24 h.

### Irradiation Procedure

Irradiation was performed using 6 MV X-ray from a linear accelerator with a dose rate of 200 monitor units per minute, delivering doses of 2, 4, and 8 Gy.

### Clonogenic Survival Assay

U87MG and GBM3 cells were treated or not with AuNPrs for 24 h. The cells were then exposed to different doses of irradiation (2 Gy, 4 Gy, and 8 Gy). 24 h after IR, the cells were detached, seeded in 6-well plates at a density of 1× 10^3^ cells/plate, and incubated in a CO_2_ incubator at 37 °C for 14 days for the growth of macroscopic colonies (50 or more cells) starting from the surviving cells. Colonies were then fixed for 15 min at room temperature using a fixation solution consisting of methanol and acetic acid (10:1, v/v) and incubated at room temperature for ∼2 h with 0.5% crystal violet in 20% methanol to allow cell staining.

For each well, we counted colonies containing ≥50 cells; the number of colonies for each radiation dose was averaged. We calculated the plating efficiency (PE) as the ratio between the number of colonies observed and the number of cells seeded. The surviving fraction (SF) was determined as colonies counted/cells plated X (PE/100). The formula “dose with radiation alone/dose with radiation + drug or radiation + AuNPrs for the same biological effect” was used to calculate the sensitizer enhancement ratio (SER). The addition of a drug or AuNPrs functions as a radiosensitizer if the SER is greater than one; if the SER value is less than one, then the compound acts as a radioprotector. All assays were performed in duplicate in at least three independent experiments.

### ATP Assay

ATP levels were evaluated using the CellTiter-Glo Luminescent Cell Viability Assay kit (Promega Italia s.r.l), following the manufacturer’s instructions. Briefly, 2.5 × 10^4^ cells were seeded in 96-well plates. After incubation for 30 min at room temperature, CellTiter-Glo Reagent was added to each well at a volume of 100 μL, contents were mixed for ∼2 min to allow cell lysis, and finally the plate was incubated at room temperature for 10 min. The luminescent signal was measured by BioTek’s Synergy HT Luminometer (BioTek Instruments Inc.).

### Confocal Microscopy

0.2 mg/mL of AuNPrs were added to U87MG cell culture in 24-well plates with round glass coverslips to assess their cellular internalization ability. After 24 h, 3.7% paraformaldehyde was used to fix cells (10 min at room temperature), 0.2% Triton X-100 for permeabilization (5 min at room temperature), and 0.4% bovine serum albumin (BSA, Sigma) in PBS for blocking (15 min at room temperature). AuNPrs were modified with the TAMRA fluorophore as a label for qualitative fluorescence imaging of NPs that have been internalized by cells, while nuclei were stained with Hoechst (Life Technologies Corporation). Finally, glass coverslips were washed with PBS and mounted on the slides. High-resolution images were acquired with a Zeiss LSM 700 laser scanning confocal microscope equipped with a Plan-Apochromat 63*x*/1.40 Oil DIC M27 immersion objective.

### Flow Cytometry Analysis

Cell death analysis was conducted using Annexin V and propidium iodide (PI) staining (Annexin V:FITC Assay Kit, Bio-Rad). Cells were grown in p60 plates at a density of 5 × 10^5^ cell/cm^2^ in supplemented DMEM, treated with 0.2 mg/mL of AuNPrs for 24 h, and subsequently irradiated at 2, 4, and 8 Gy. 24 h after IR, the cells were collected and resuspended in Annexin V binding buffer (10 mM HEPES/NaOH, pH 7; 140 mM NaCl; 2.5 mM CaCl2). Following the manufacturer’s protocol, the samples were stained first with 5 μL of Annexin V-FITC for 20 min and then with 10 μL of PI, followed by flow cytometry analysis. 10.000 events were collected by a flow cytometer, and BD Accuri C6 Software was used for data analysis. Cells positive for Annexin V only were identified as apoptotic cells.

### Statistical Analysis

GraphPad prism 7.0 software for Windows (GraphPad software) was used for the statistical analysis of the obtained data. Data are presented as mean ± SD and were analyzed using two-tailed Student’s *t*-test or one-way ANOVA for independent groups, followed by Bonferroni correction for multiple comparisons. Statistical significance was defined as *P* < 0.05.

## Results

### AuNPrs Effects in GBM Cells

Nanotechnology has been gaining more and more attention for biomedical applications and has already been studied for the optimization of radiosensitization in cancer cells. Indeed, AuNPs, given their physical and chemical properties, have shown promising potential in improving GBM susceptibility to radiotherapy.^6^ First, we performed a cell viability assay to evaluate the toxicity of specifically shaped AuNPs, gold nanoprisms (AuNPrs) (Figure 1A–D), in U87MG and GBM3 cell lines at different concentrations. In order to do so, we measured cytotoxicity using an MTT assay after treatment with AuNPrs at 0.1, 0.2, 0.4, or 0.8 mg/mL for 24 h. As seen in Figure 1, AuNPrs have no cytotoxic effect on GBM cells (Figure 1E). This is in line with other reports using these AuNPrs in different model systems.^12^ Considering that the concentration of 0.2 mg/mL, one of the lowest tested, did not result in cellular toxicity and, as shown in other reports,^10^ was sufficient for cellular uptake, the following experiments were performed using this dose. Consistently, fluorescence microscopy analysis of GBM cells treated with AuNPrs labeled with TAMRA at 0.2 mg/mL revealed red signals within the cytoplasm^13^ (Figure 2).

**Figure 1:**
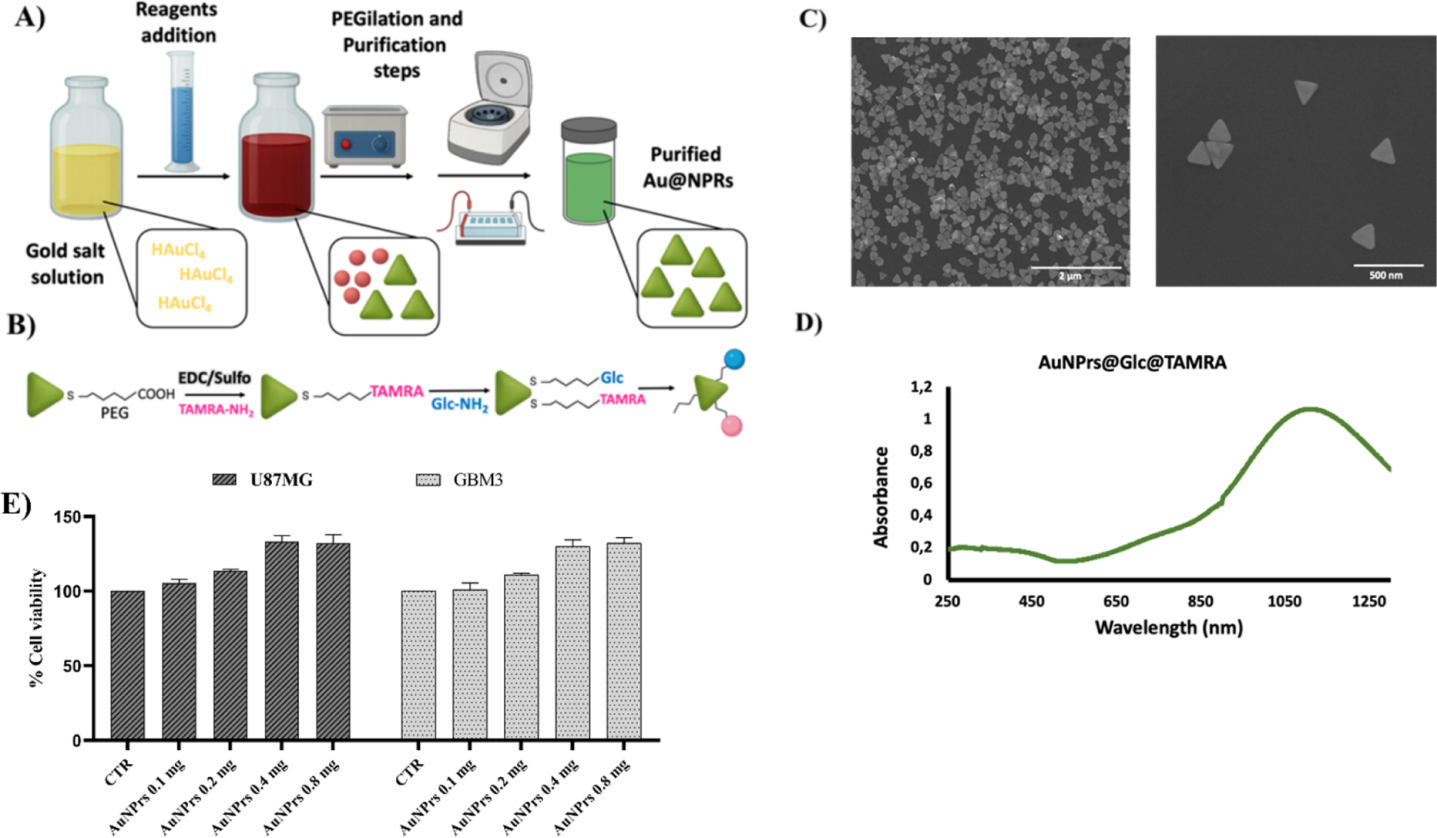
AuNPrs have no toxic effects on GBM cells. (A) Schematic representation of the synthetic procedures for gold nanoprisms (AuNPr) used for glioblastoma treatment and (B) the diagram of EDC/NHS-mediated covalent coupling of PEG-derived AuNPrs with TAMRA and amine-modified glucose. Characterization of the AuNPrs was detailed in (C), where SEM images show an average particle size of 210 ± 46 nm. (D) the UV–vis spectra show an LSPR band at 1100 nm. (E) AuNPrs (from 0.1 mg/mL to 0.8 mg/mL) were added to GBM cell cultures to evaluate their impact on cell viability. The data shows that AuNPrs have no toxic effects on U87MG and GBM3 cells. No statistically significant differences were observed (ns). All data are presented as the mean ± SD of at least three independent experiments.

**Figure 2:**
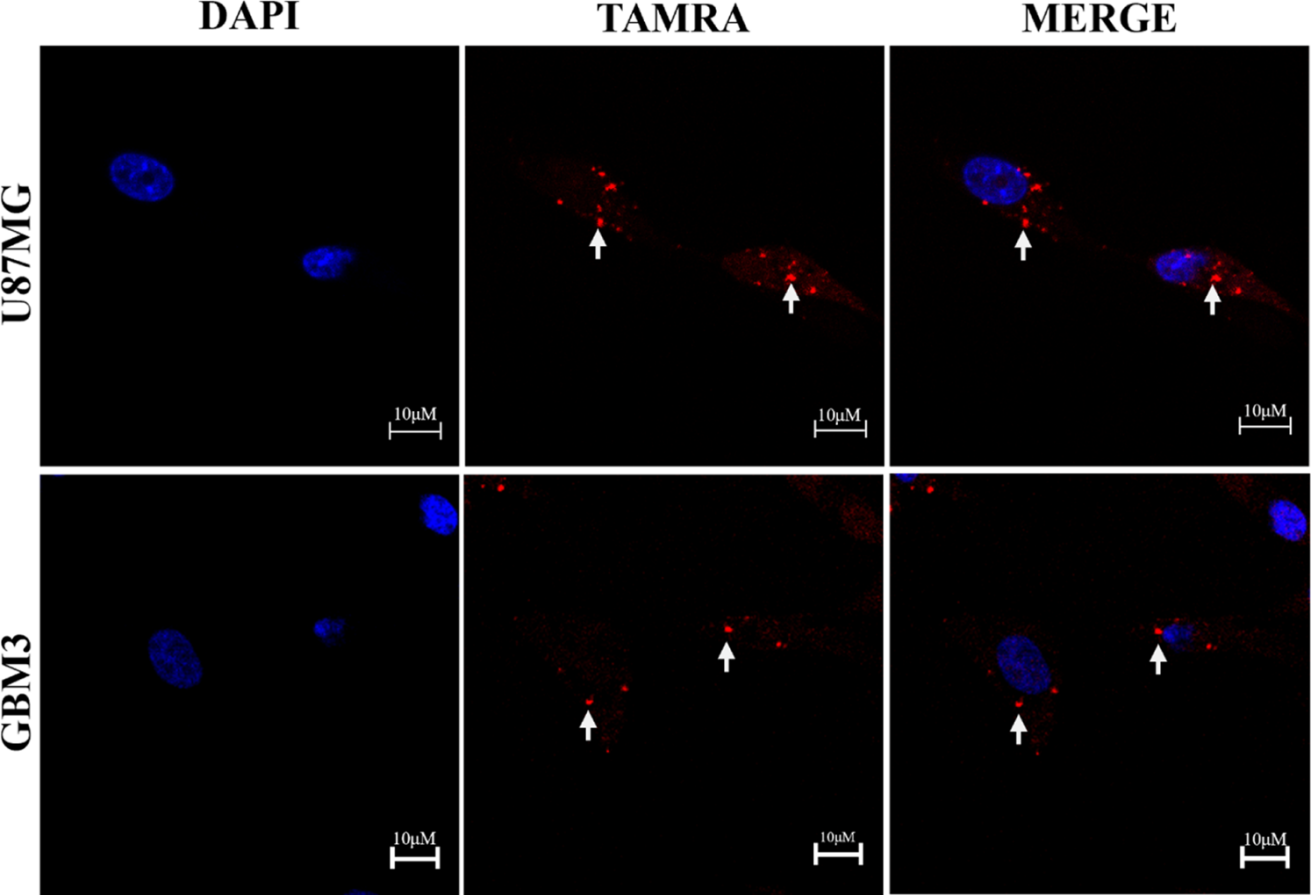
AuNPrs uptake in GBM cells. AuNPrs modified with the TAMRA fluorophore (red) and nuclei with DAPI (blue). Red fluorescence staining was detected in the cell cytoplasm, indicating a cytoplasmic location for the AuNPrs. White arrows highlight the sites of AuNPrs internalization within the cells.

### AuNPrs Combined with IR Improve the Radiosensitivity of GBM Cells

Next, we examined the effect of AuNPrs on the radiosensitivity of GBM cells through a colony formation assay. A group of GBM cells were treated with ionizing radiation (IR) alone at 2, 4, 6, and 8 Gy for few minutes, while another group of GBM cells were pretreated with 0.2 mg/mL AuNPrs for 24 h and then exposed to ionizing radiation (IR) above described. After 24 h at 37 °C, the clonogenic assay was carried out. In the U87MG cell line and primary GBM3 cell line, the addition of AuNPrs markedly increased sensitivity to IR, as evidenced by the reduced colony-forming ability when compared to IR alone (Figure 3A,B). We observed an IR-dose dependent reduction of the clonogenic survival of GBM cells treated with AuNPrs + IR (Figure 3A,B). The extent of radiosensitization was quantified by comparing the surviving fractions at the dose of 2 Gy (SF2) (Figure 3C) and by assessing the sensitizer enhancement ratios (SER) (Figure 3D).^14^ The addition of AuNPrs decreased the SF2 values of GBM cells compared to cells exposed to IR alone. In particular, the combined treatment reduced the SF2 values of U87MG cells from 82.5% ± 2.2 to 54.2% ± 0.5 and of GBM3 cells from 84.4% ± 1.1 to 56.7% ± 1.2 (Figure 3C). The SER values, calculated at 50% cell survival as “dose with irradiation alone/dose of irradiation with nanoparticles”, showed values greater than 1 in both cell lines, specifically, 2.33 for U87MG and 1.6 for GBM3, suggesting that the AuNPrs could have a radiosensitizing effect (Figure 3D). Moreover, to confirm these results, we performed an MTT assay 24 h post radiation of GBM cells treated with AuNPrs + IR and IR alone. We observed that the combined treatment inhibited the viability of GBM cells to a greater extent than those treated with IR alone (Figure 3E). Finally, it is worth noting that neither the combined treatment nor radiation alone had significant effects on the viability of human astrocytic cells, NHA cells, which represent the healthy counterpart of the brain (Figure 3E).

**Figure 3:**
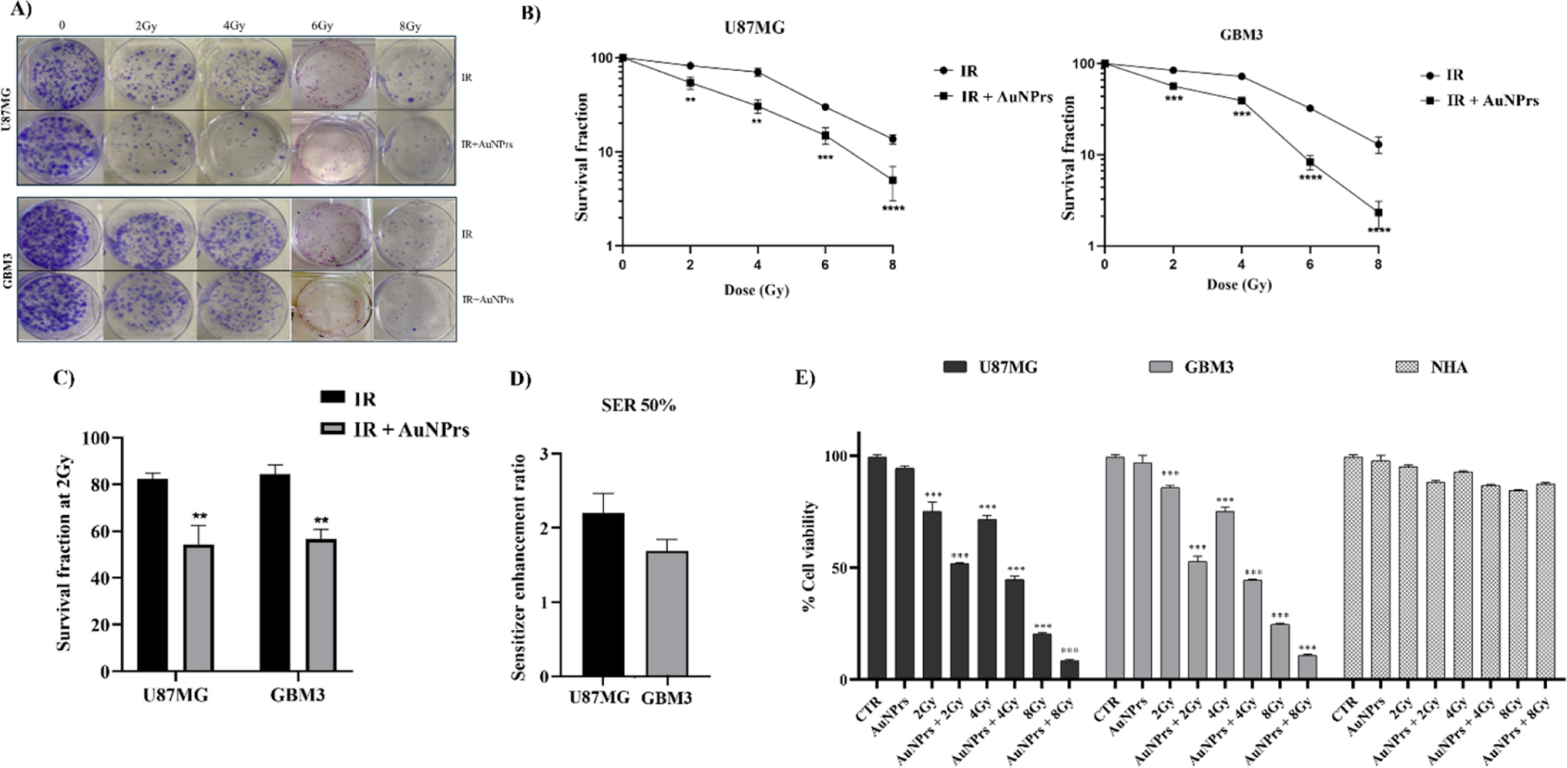
Radiosensitizing effects of AuNPrs. (A) U87MG and GBM3 cells were treated with AuNPrs (0.2 mg/mL) and irradiated after 24 h with 2 Gy, 4 Gy, 6 Gy, and 8 Gy doses. After irradiation treatment, the cells were seeded in 6-well plates (1 × 10^3^ cells/plate) and were grown for 14 days. (B) Colony were then counted for each well, and the number of colonies corresponding to each radiation dose was averaged. The results shown represent the average of 3 independent experiments. (ANOVA. **p < 0.01, ***p < 0.001, and ****P < 0.0001 compared to the control group; **p < 0.01, ***p < 0.001, and ****P < 0.0001 when the combination of AuNPrs + IR was compared to IR alone.). (C) Representative graph for the survival fraction at 2 Gy (SF2) of glioblastoma lines treated with AuNPrs + IR or IR alone (ANOVA. **P < 0.01 when the combination of AuNPrs + IR was compared to IR alone). (D) SER was calculated at 50% cell survival as “dose with radiation alone/dose with radiation + AuNPrs”. The SER obtained was 2.33 for U87MG and 1.6 for GBM3. Since the value is greater than 1 in both cell lines, the addition of AuNPrs worked as a radiosensitizer. (E) GBM cells after incubation with 0.2 mg/mL AuNPrs, were exposed to IR at 2 Gy, 4 Gy, and 8 Gy doses. After 24 h, they were plated in 96-well plates (1× 10^3^ cells/well) to perform the MTT assay. The combination treatment with IR + AuNPrs has been shown to reduce cell viability compared to IR alone in both GBM cell lines. The combined treatment with IR + AuNPrs has not shown effects on NHA cells. The results shown represent the average of 3 independent experiments. ANOVA ***p < 0.001 compared to the control group; ***p < 0.001 when the combination of AuNPrs + IR was compared to IR alone.

### AuNPrs-Induced Direct Cell Death

To characterize the molecular mechanism of AuNPrs-mediated radiosensitization, we performed flow cytometric analysis of the cell death mechanism induced by the combined IR and AuNPrs treatment. In order to evaluate if the AuNPrs + IR combination could trigger apoptosis or necrosis in GBM cells, we performed analysis by Annexin V and propidium iodide (PI) double staining. We chose 2 Gy as a representative dose, as it was the lowest dose at which a significant radiosensitizing effect was still observed. Notably, we observed an IR dose-dependent induction of necrosis (AV^–^/PI^+^) and late apoptosis (AV^+^/PI^+^) in samples treated with AuNPrs + IR compared with IR alone. The rate of necrosis was higher than the rate of late apoptosis, suggesting that the mechanism of the radiosensitizing effect of AuNPrs may involve non-apoptotic cell death mechanisms and is most likely related to direct cell death by necroptosis (Figure 4).

**Figure 4:**
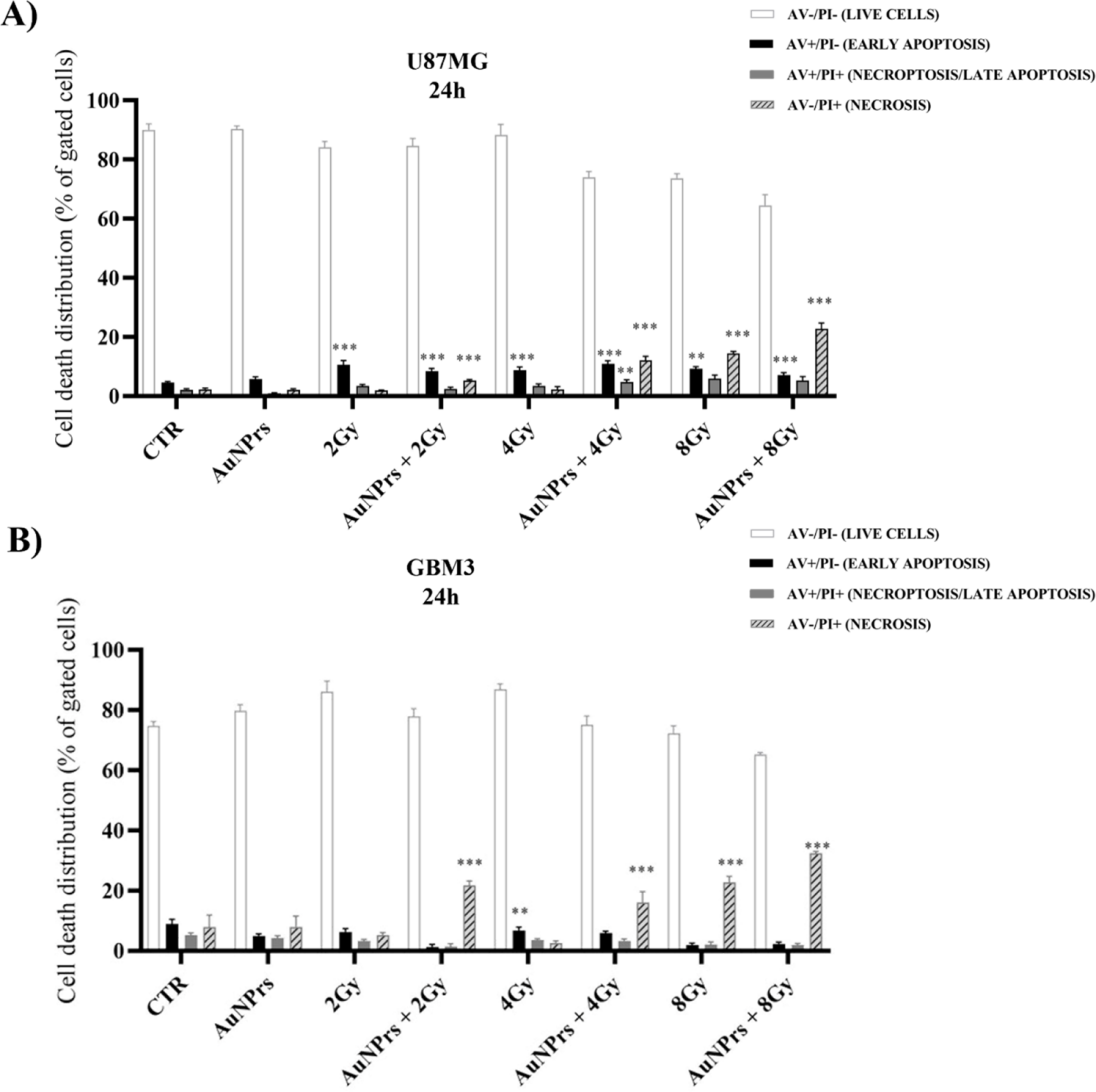
Effects of AuNPrs + IR on cell death. Flow cytometry images representing apoptosis and necrosis induction rates of U87MG cells (A) and GBM3 (B) combined with AuNPrs + IR or radiation alone. AuNPrs, 0.2 mg/mL, were added to cell cultures and irradiated with doses of 2, 4, and 8 Gy; samples were collected after 24 h and then analyzed. The images show an increase of the percentage of necrotic cells following AuNPrs + IR treatment compared to apoptotic cells after treatment with radiation alone. The results shown represent the average of 3 independent experiments. The apoptosis rate was calculated and depicted in a bar chart, and the statistical values were determined using ANOVA (**p < 0.01; ***p < 0.001 compared to the control group; **p < 0.01; ***P < 0.001 when the combination of AuNPrs + IR was compared to IR alone).

We then investigated the underlying cell death mechanism at the molecular level in the U87MG cell line to confirm our hypothesis. To elucidate the pathways involved, we conducted molecular characterization experiments, including the assessment of intracellular ATP depletion and the activation of necroptosis markers. Regarding the first point, a significant reduction of the intracellular ATP levels was noted in cells treated with AuNPrs + IR at 6 h compared with cells treated with IR alone (Figure 5A). Necroptosis is a programmed cellular death pathway, dependent on the formation of the RIP1/RIP3/MLKL necrosome complex.^15^ Since RIP1, RIP3, and MLKL are the key effectors of the necroptosis signaling pathway, we analyzed their expression,^15^ by means of the qRT-PCR assay. We observed an increase in necroptosis markers in U87MG cells treated with AuNPrs + IR 2 Gy compared to cells treated with IR 2 Gy alone. The activation of these proteins is associated with the increase of HMGB1 and PUMA expression which has recently been linked to the execution of necroptosis^15^ (Figure 5B). Figure S1 (Supporting Information) shows the plots of the flow cytometry analysis.

**Figure 5:**
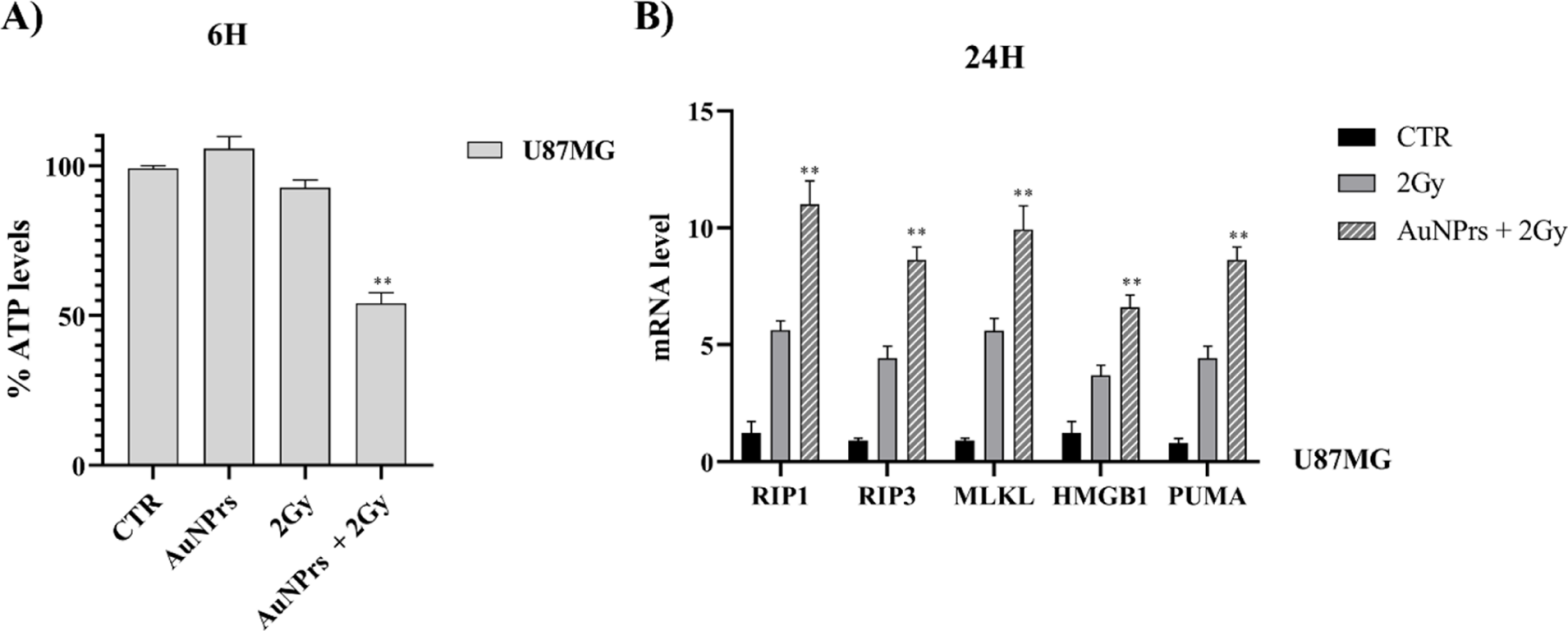
Effects of AuNPrs + IR on ATP levels and expression of necroptosis markers. AuNPrs (0.2 mg/mL) were added to cell cultures and irradiated with a dose of 2 Gy; after 6 h, the ATP assay (A) and, after 24 h, RT-qPCR (B) analysis were performed. Treatment with AuNPrs + IR significantly reduced the ATP levels and increased the mRNA expression of necroptosis-related markers including RIP1, RIP3, MLKL, HMGB1, and PUMA. The results shown represent the average of 3 independent experiments (ANOVA; **P < 0.01 compared to the control group; **P < 0.01 when the combination of AuNPrs + IR was compared to IR alone).

## Discussion

Radiotherapy (RT) is one of the most effective and widely used therapies for cancer. The current standard of care for GBM consists of surgical resection, followed by RT in combination with temozolomide (TMZ) [Stupp protocol].^3^ RT treatment of GBM consists of daily fractions of 2 Gy given 5 days per week for 6 weeks for a total treatment of approximately 60 Gy^2^. The goal of RT is to deliver the maximum dose to the tumor tissue while saving the surrounding normal tissue. However, normal tissue cytotoxicity and tumor RR remain critical problems in RT. In fact, most tumors relapse locally after RT, which has driven the search for novel therapeutic strategies aimed at enhancing tumor radiosensitivity and ultimately improving the efficacy of RT. Recent studies have reported encouraging results in the application of nanoparticles as a promising strategy to improve radiotherapeutic efficacy in cancer treatment. Among the various nanoplatforms investigated for radiotherapeutic applications, gold nanoparticles (AuNPs) have been studied most extensively due to their high X-ray absorption coefficient, as well as ease of synthesis.^16^ In fact, extensive preclinical studies have reported that AuNPs significantly enhanced the local radiation dose and increased radiation-induced cytotoxicity across various cancer types, including GBM.^17^ For example, Butterworth et al.^18^ observed that AuNPs increased the efficacy of RT at 4 Gy in T98G cells, as shown by an SER of 1.85; this highlights the broad radiosensitizing potential of AuNPs in brain cancer models.

In this study, we report the use of gold nanoprisms (AuNPrs), anisotropic AuNPs, as radiosensitizing agents not previously evaluated in GBM cells. AuNPrs have been used as photothermal agents for photothermal therapy (PTT) for several cancer cells, thanks to their ability to absorb near-infrared wavelengths and convert the absorbed energy into heat, but are not yet explored for RT enhancement in GBM models. Such metallic nanoparticles with anisotropic geometries are known to exhibit physicochemical properties distinct from the most commonly used spherical particles, with flat facets and sharp edges contributing to enhanced energy absorption.^19,20^ Their biological effects at the cellular level remain less well characterized, highlighting the need for exploratory studies such as that in the present work. In this context, these characteristics provide one of the main rationales for evaluating AuNPrs as candidate radiosensitizers in GBM.

Indeed, the present study shows that low concentrations (0.2 mg/mL) of AuNPrs enhance the radiosensitivity of GBM cells. Our findings demonstrate that AuNPrs are non-toxic to the two cellular models here examined, the immortalized cell line U87MG and the primary cell line GBM3, at the tested concentrations (from 0.1 to 0.8 mg/mL, Figure 1), exhibiting efficient cellular internalization (Figure 2). AuNPrs, supplied at 0.2 mg/mL on all of the following experiments, helped increase radiation sensitivity in both cell lines, as shown by the cell viability assay and the clonogenic assay (Figure 3). Indeed, AuNPrs in combination with a low dose of ionizing radiation (2 Gy) inhibited their colony forming ability. Moreover, the reduction in SF at 2 Gy values and an SER at 2.33 for U87MG and 1.6 for GBM3, compared to cells treated with IR alone, suggest a radiosensitizing effect of AuNPrs (Figure 3). Thus, the usage of AuNPrs in RT could offer several benefits, such as reducing the doses of both agents to minimize side effects while expanding the therapeutic window of RT. Interestingly, we employed NHA as a non-tumor control to evaluate the tumor selectivity of AuNPrs-mediated radiosensitization; indeed, our results show that AuNPrs selectively radiosensitize GBM tumor cells while avoiding effects on normal astrocytes.

These encouraging data prompted us to further characterize the molecular effects of the combined treatment of AuNPrs and low-dose IR. Therefore, we explored whether the AuNPrs + IR combination could induce apoptotic or necrotic cell death by performing Annexin V/PI double staining, followed by flow cytometry analysis. Flow cytometry results showed that AuNPrs pretreatment promoted a form of cell death named necroptosis, a regulated necrotic- and caspase-independent form of cell death.^21^ In line with this observation, the combined AuNPrs + IR treatment (2 Gy) led to an upregulation of RIP1, RIP3, and MLKL mRNA compared with cells exposed to IR alone. These markers are key components of the necrosome complex and are required for the execution of necroptosis.^21^ Necroptosis is started by RIP1 activation, which binds to RIP3 and promotes its oligomerization and autophosphorylation. Subsequently, MLKL is phosphorylated by RIP3 and translocates to cellular membranes, where it contributes to membrane permeabilization and cell lysis. This process is also characterized by the extracellular release of HMGB1 as a consequence of membrane permeabilization,^22^ as well as mitochondrial dysfunction and ATP depletion.^23^ Indeed, here, we report that GBM cells treated with AuNPrs + IR at 2 Gy display an upregulation of HMGB1 mRNA and a marked reduction of ATP levels, in line with previous reports identifying ATP depletion as a key biochemical hallmark of necroptosis. Remarkably, the observed ATP depletion possibly reflects an early metabolic effect occurring at 6 h post treatment prior to detectable cell loss, rather than a secondary consequence of reduced cell viability. Moreover, since PUMA-increased expression has been linked to multiple necroptosis-related diseases, including ischemia-reperfusion,^24^ and is mediated by MLKL activation, we also analyzed PUMA expression in GBM cells treated with AuNPrs + IR 2 Gy combo. We observed an upregulation of PUMA mRNA, supporting its involvement in the execution of necroptosis. On this note, He et al. showed that the combination of RT and AuNPs can induce a stronger effect of immunogenic cell death (ICD) on GBM cells compared to the use of RT alone, resulting in the release of damage-associated molecular patterns (DAMPs) such as HMGB1 and ATP.^25^

Taken together, these findings support the advantages regarding the use of AuNPrs as radiosensitizers for the GBM. A number of AuNPs formulations have already reached clinical trials for cancer treatment, including CYT-6091 (citrate-coated AuNPs bound with thiolated PEG and TNFalpha) and NU-0129 (Spherical Nucleic Acid AuNP).^26^ Although the radiosensitizing effects of gold-based nanomaterials in GBM have been widely investigated,^27^ this study represents, to the best of our knowledge, the first report of AuNPrs being evaluated as radiosensitizers in GBM cellular models. In this context, our results show that the combination of AuNPrs with low-dose IR (2 Gy) enhances radiosensitivity and influences the mode of cell death, promoting a regulated necrotic program consistent with necroptosis. In particular, the AuNPrs + IR combination, effective as demonstrated by an SER value >1 and the induction of a form of immunogenic cell death, suggests the possibility of using this approach to improve the specificity of RT treatment currently in use, strengthening the relevance of these results in the context of GBM.

## Conclusions

Gold nanoprisms (AuNPrs), when combined with 2 Gy of IR, remarkably radiosensitize GBM cells, reducing cell viability and colony formation ability, and, most importantly, trigger necroptotic cell death when compared with samples treated with 2 Gy alone. It is important to note that this study has been carried out on in vitro models, so the implications for clinical translation are still preliminary. There are different key future challenges to consider, such as administration and blood–brain barrier penetration as well as long-term persistence in brain tissue. Overall, further in vivo studies are needed to evaluate these aspects and to fully understand the translational potential of AuNPrs. Nevertheless, these data remain encouraging, highlighting the need for further testing in animal models, as well as future clinical trials.

## Supplementary Material



## Data Availability

All data generated or analyzed during this study are included in this published article [and its Supporting Information files].

## Notes

Figure S1 Flow cytometry plots showing the gating strategy and proportion of U87MG (A) and GBM3 (B) cells for apoptosis analysis with double staining Annexin V–PI (PDF)

## List of Abbreviations

ABM: astrocytes basal medium
AuNPrs: gold nanoprisms
AuNPs: gold nanoparticles
BSA: bovine serum albumin
DMEM: Dulbecco’s modified Eagle’s medium
FBS: fetal bovine serum
GBM: glioblastoma multiforme
GSCs: glioblastoma stem cells
ICD: immunogenic cell death
IR: ionizing radiation
MTT: 3-[4,5-dimethylthiazol-2-yl]-2,5 diphenyl tetrazolium bromide
NHA: normal human astrocytes
PBS: phosphate-buffered solution
PE: plating efficiency
PEG: polyethylene glycol
RR: radioresistance
RT: radiotherapy
SER: sensitization enhancement ratio
SF: surviving fraction
SF2: surviving fractions at a radiation dose of 2 Gy
TAMRA: tetramethylrhodamine 5 (6)-carboxamide cadaverine
TMZ: temozolomide

## References

[1] Hambardzumyan D., Bergers G. (2015). Glioblastoma: Defining Tumor Niches. Trends Cancer.

[2] Uhm J. H., Porter A. B. (2017). Treatment of Glioma in the 21st Century:
An Exciting
Decade of Postsurgical Treatment Advances in the Molecular Era. Mayo Clin. Proc..

[3] Stupp R., Mason W. P., van den Bent M. J. (2005). Radiotherapy plus concomitant
and adjuvant Temozolomide for glioblastoma. N. Engl. J. Med..

[4] Wu W., Klockow J. L., Zhang M. (2021). Glioblastoma multiforme
(GBM): An overview of current therapies and mechanisms of resistance. Pharmacol. Res..

[5] Wu Y., Song Y., Wang R., Wang T. (2023). Molecular mechanisms
of tumor resistance to radiotherapy. Mol. Cancer.

[6] Ruiz-Garcia H., Ramirez-Loera C., Malouff T. D., Seneviratne D. S., Palmer J. D., Trifiletti D. M. (2021). Novel Strategies
for Nanoparticle-Based
Radiosensitization in Glioblastoma. Int. J.
Mol. Sci..

[7] Her S., Jaffray D. A., Allen C. (2017). Gold nanoparticles
for applications
in cancer radiotherapy: Mechanisms and recent advancements. Adv. Drug Deliv. Rev..

[8] Ruchika F., Suvarnapathaki S., Serrano-Farias A., Bettegowda C., Rincon-Torroella J. (2025). GLUT1 as a
Potential Therapeutic Target in Glioblastoma. Brain Sci..

[9] Alfranca G., Artiga A. ´., Stepien G., Moros M., Mitchell S. G., de la
Fuente J. M. (2016). Gold nanoprism-nanorod face off: comparing the heating
efficiency, cellular internalization and thermoablation capacity. Nanomedicine.

[10] Moros M., Lewinska A., Merola F. (2020). Gold Nanorods and Nanoprisms
Mediate Different Photothermal Cell Death Mechanisms In Vitro and
In Vivo. ACS Appl. Mater. Interfaces.

[11] Pagano C., Navarra G., Pastorino O. (2021). N6-Isopentenyladenosine
Hinders the Vasculogenic Mimicry in Human Glioblastoma Cells through
Src-120 Catenin Pathway Modulation and RhoA Activity Inhibition. Int. J. Mol. Sci..

[12] Pérez-Hernández M., Moros M., Stepien G., del Pino P., Menao S., de las Heras M., Arias M., Mitchell S. G., Pelaz B., Gálvez E. M. (2017). Multiparametric analysis of anti-proliferative
and apoptotic effects of gold nanoprisms on mouse and human primary
and transformed cells, biodistribution and toxicity in vivo. Part. Fibre Toxicol..

[13] Baffou G., Bon P., Savatier J. (2012). Thermal imaging of nanostructures by quantitative
optical phase analysis. ACS Nano.

[14] Navarra G., Pagano C., Pacelli R., Crescenzi E., Longobardi E., Gazzerro P., Fiore D., Pastorino O., Pentimalli F., Laezza C. (2020). N^6^-Isopentenyladenosine
Enhances the Radiosensitivity of Glioblastoma Cells by Inhibiting
the Homologous Recombination Repair Protein RAD51 Expression. Front. Oncol..

[15] Pagano C., Navarra G., Coppola L., Avilia G., Pastorino O., Della Monica R., Buonaiuto M., Torelli G., Caiazzo P., Bifulco M. (2022). N6-isopentenyladenosine induces cell death through
necroptosis in human glioblastoma cells. Cell
Death Discov..

[16] Choi J., Kim G., Cho S. B., Im H. J. (2020). Radiosensitizing high-Z metal nanoparticles
for enhanced radiotherapy of glioblastoma multiforme. J. Nanobiotechnol..

[17] Qureshi S., Anjum S., Hussain M. (2024). A recent insight of
applications of gold nanoparticles in glioblastoma multiforme therapy. Int. J. Pharm..

[18] Butterworth K. T., Coulter J. A., Jain S. (2010). Evaluation of cytotoxicity
and radiation enhancement using 1.9 nm gold particles: potential application
for cancer therapy. Nanotechnology.

[19] Joshi R., Sharma S., Ali N., Muteeb G. (2025). Harnessing
the potential of nanoprisms for diagnostic and therapeutic applications. Colloid Interface Sci. Commun..

[20] Gonnelli A., Gerbé de Thoré M., Ermini M. L., Frusca V., Zamborlin A., Signolle N., Bawa O., Clémenson C., Meziani L., Bergeron P. (2024). Nonpersistent Nanoarchitectures
Enhance Concurrent Chemoradiotherapy in an Immunocompetent Orthotopic
Model of HPV+ Head/Neck Carcinoma. Adv. Mater..

[21] Zhang T., Wang Y., Inuzuka H., Wei W. (2022). Necroptosis pathways
in tumorigenesis. Semin. Cancer Biol..

[22] Chen R., Kang R., Tang D. (2022). The mechanism
of HMGB1 secretion
and release. Exp. Mol. Med..

[23] Eguchi Y., Shimizu S., Tsujimoto Y. (1997). Intracellular
ATP levels determine
cell death fate by apoptosis or necrosis. Cancer
Res..

[24] Wu B., Qiu W., Wang P. (2007). p53 independent induction of PUMA mediates
intestinal apoptosis in response to ischaemia-reperfusion. Gut.

[25] He C., Ding H., Li L. (2023). Gold Nanoparticles Enhance
the Ability of Radiotherapy to Induce Immunogenic Cell Death in Glioblastoma. Int. J. Nanomed..

[26] Joh D. Y., Sun L., Stangl M. (2013). Selective targeting of brain tumors with gold
nanoparticle-induced radiosensitization. PLoS
One.

[27] Guerra D. B., Oliveira E. M. N., Sonntag A. R., Sbaraine P., Fay A. P., Morrone F. B., Papaléo R. M. (2022). Intercomparison
of radiosensitization
induced by gold and iron oxide nanoparticles in human glioblastoma
cells irradiated by 6 MV photons. Sci. Rep..

