# Supplementary material for: Gold Nanoparticles Enhance Radiosensitivity in Glioblastoma Cells
Source: ACS Omega. 2026 Mar 17;11(12):19839–47. doi: 10.1021/acsomega.6c00540 (PMC13044645; doi:10.1021/acsomega.6c00540)

SUPPORTING INFORMATION

Gold nanoparticles enhance radiosensitivity in glioblastoma cells.

Laura Coppola[1], Giovanna Navarra[1], Giorgio Avilia[1], Carlos Cuestas-Ayllón[2,3], María Moros[2,3], Jesús Martínez de la Fuente[2,3], Pasqualino De Antonellis[1], Maria Teresa Gentile[4], Laura Mosca[5], Caterina Oliviero[6], Roberto Pacelli[7], Chiara Laezza[8], Maurizio Bifulco[1 †*] and Cristina Pagano[1 †*].

[1]Department of Molecular Medicine and Medical Biotechnology, University of Naples “Federico II”, 80131 Naples, Italy

[2]Instituto de Nanociencia y Materiales de Aragón (INMA), CSIC-Universidad de Zaragoza, 50009 Zaragoza, Spain

[3]Centro de Investigación Biomédica en Red de Bioingeniería, Biomateriales y Nanomedicina (CIBER-BBN), 28029 Madrid, Spain;

[4]Department of Environmental, Biological and Pharmaceutical Sciences and Technologies (DiSTABiF), University of Campania 'Luigi Vanvitelli', 81100 Caserta, Italy

[5]Department of Human Sciences and Promotion of the Quality of Life, San Raffaele University, 00166 Rome, Italy.

[6]Unit of Medical Physics and Radioprotection, University Hospital Federico II, 80131 Naples, Italy

[7]Department of Advanced Biomedical Sciences, University Federico II, 80131 Naples, Italy

[8]Institute of Endocrinology and Experimental Oncology (IEOS), National Research Council (CNR), 80131 Naples, Italy

*Corresponding author: Correspondence to Maurizio Bifulco (maubiful@unina.it) or Cristina Pagano (cristina.pagano@unina.it)

†These authors are co-last authors.

This SI file contains 1 page with Figure S1.

Figure S1 Flow cytometry plots showing the gating strategy and proportion of U87MG (A) and GBM3 (B) cells for apoptosis analysis with double staining Annexin V - PI.

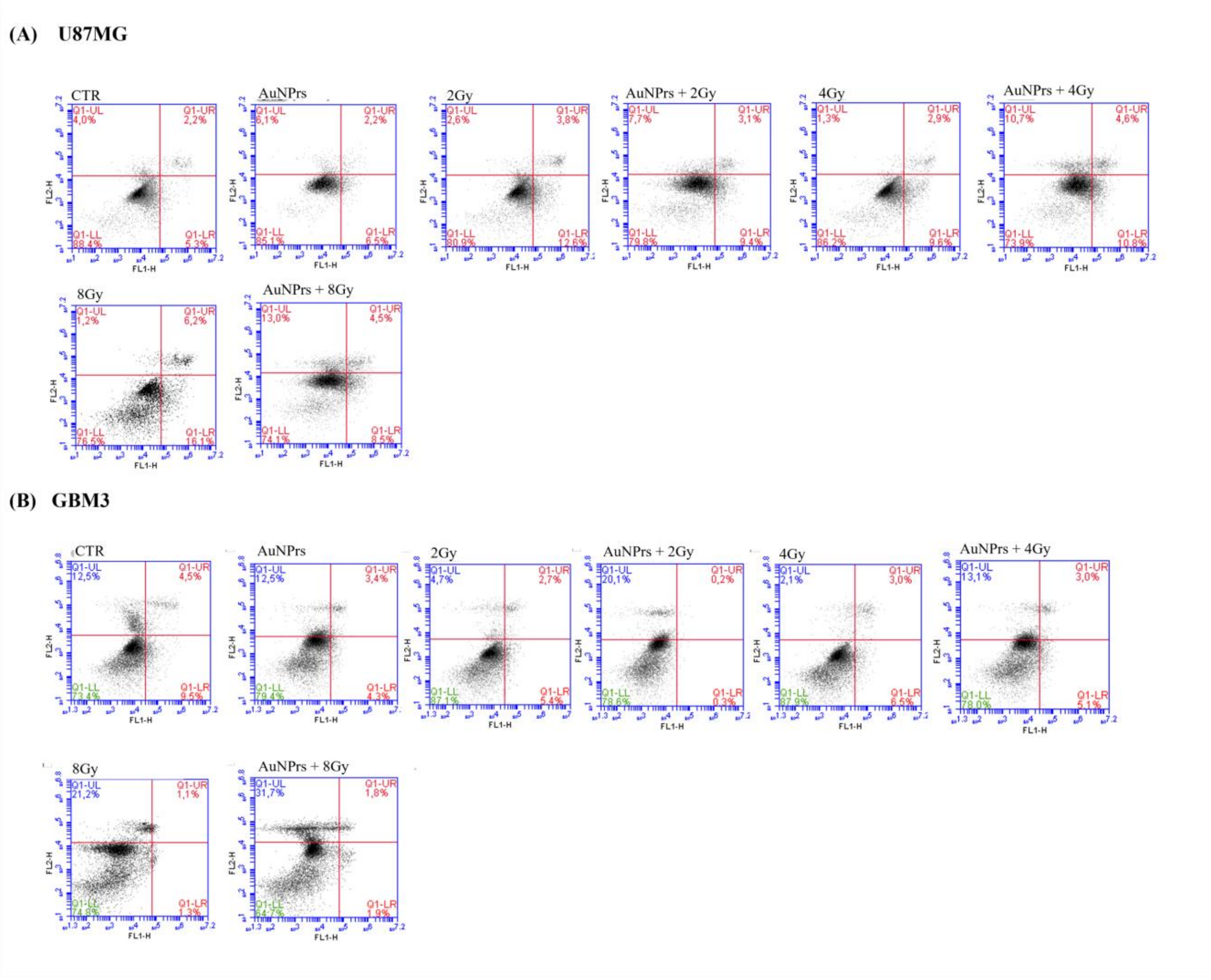

Supplement: Supplementary file 1 [file ao6c00540_si_001.pdf]
